# Using a Novel MicroRNA Delivery System to Inhibit Osteoclastogenesis

**DOI:** 10.3390/ijms16048337

**Published:** 2015-04-14

**Authors:** Yanlan Yao, Tingting Jia, Yang Pan, Hongna Gou, Yulong Li, Yu Sun, Rui Zhang, Kuo Zhang, Guigao Lin, Jiehong Xie, Jinming Li, Lunan Wang

**Affiliations:** 1 National Center for Clinical Laboratories, Beijing Hospital, No. 1 Dahua Road, Dongdan, Beijing 100730, China; E-Mails: yaoylan@126.com (Y.Y.); jtt216@126.com (T.J.); gogo2026@126.com (H.G.); 13552566721@139.com (Y.L.); ysunchina@163.com (Y.S.); zrzxd@sina.com (R.Z.); sarahkuo@163.com (K.Z.); linguigao1982@163.com (G.L.); jhxie@nccl.org.cn (J.X.); 2 Graduate School, Peking Union Medical College, Chinese Academy of Medical Sciences, No. 9 Santiao, Dongdan, Beijing 100730, China; 3 Institute for Infectious Disease and Endemic Disease Control, Beijing Center for Disease Prevention and Control (CDC), Beijing 100013, China; E-Mail: panyang10@gmail.com

**Keywords:** MS2 VLP, miR-146a, rheumatoid arthritis, osteoporosis, osteoclast

## Abstract

Previously, we developed a novel microRNA (miRNA) delivery system based on bacteriophage MS2 virus-like particles (MS2 VLPs). In this current study, we used this system to transport miR-146a into human peripheral blood mononuclear cells (PBMCs), and demonstrated the inhibition of osteoclastogenesis in precursors. Two cytokines, receptor activator of NF-κB ligand (RANKL), and macrophage-colony stimulating factor (M-CSF) were used to induce osteoclastogenesis. MS2 VLPs were transfected into PBMCs. qRT-PCR was applied to measure expression levels of miR-146a and osteoclast (OC)-specific genes. Western blot (WB) was conducted to evaluate miR-146a downstream target proteins: epidermal growth factor receptor (EGFR) and tumor necrosis factor (TNF) receptor-associated factor 6 (TRAF6). The formation and activity of OCs were assessed by cytochemical staining and bone resorption assay, respectively. In PBMCs treated with MS2-miR146a VLPs, qRT-PCR assays showed increased expression of miR-146a (*p* < 0.01) and decreased expression of all four OC-specific genes (*p* < 0.05). WB results indicated decreased expression of EGFR (*p* < 0.01) and TRAF6 (*p* < 0.05). The number of OCs decreased markedly and bone resorption assay demonstrated inhibited activity. This miR-146a delivery system could be applied to induce overexpression of miR-146a and to inhibit the differentiation and function of OCs.

## 1. Introduction

Bone remodeling is a continuous process kept in balance by two types of cells: bone-forming osteoblasts and bone-resorbing osteoclasts (OCs). Osteoporosis is a prevalent disorder caused by a relative increase in bone resorption over bone formation [1]. Osteoporosis may also occur secondarily to rheumatoid arthritis (RA), which is characterized by chronic inflammation of synovial tissue and irreversible joint destruction [2,3]. OCs, the major cells for bone resorption, play a pivotal role in the development and progression of bone loss [3,4]. OCs are formed as multinucleate giant cells originating from the monocyte/macrophage lineage [5,6]. Theoretically, inhibition of osteoclastogenesis should benefit patients suffering from osteoporosis [3,7].

miRNAs constitute a recently discovered family of small RNAs, 21–25 nucleotides in length that participate in posttranscriptional regulation of gene expression. Some miRNAs play critical roles in immunoregulation, and are involved in the pathogenesis of osteoporosis or RA [2,8,9,10]. It is clear that miRNAs are emerging as potential targets or tools for new therapeutic strategies in the treatment and prevention of autoimmune disorders. Therapeutic trials to target miRNAs have been conducted to treat some diseases, including colon and breast cancers [11,12,13,14]. It has been reported that miR-146a, a negative regulator of autoimmunity [15,16], plays a role in osteoclastogenesis. Although the details have not been fully elucidated, several reports have proposed mechanisms to explain the role of miR-146a [17,18,19,20].

At present, the ideal delivery vehicle is the Rosetta Stone of miRNA-based molecular therapy; thus, an effective delivery method for transfection of synthetic miRNA is urgently required. The ideal delivery system is expected to transport miRNAs to the right place without being degraded by endogenous RNases. Herein, we previously developed a delivery system based on MS2 VLPs and Tat_47–57_ peptide, derived from human immunodeficiency virus 1 (HIV-1) [21,22]. In the present study, we used the monocyte/macrophage lineage of human PBMCs as OC precursors [23,24] to demonstrate that this system can be applied for the upregulation of miR-146a and inhibition of osteoclastogenesis. As expected, upregulated miR-146a displayed an inhibitory role and there was a marked decrease in the expression of downstream proteins and OC-specified marker genes. Moreover, tartrate-resistant acid phosphatase (TRAP) staining and pits formation assay also demonstrated the inhibition of OC formation and activity.

## 2. Results

### 2.1. Expression of miR-146a Increased Dramatically

**Figure 1:**
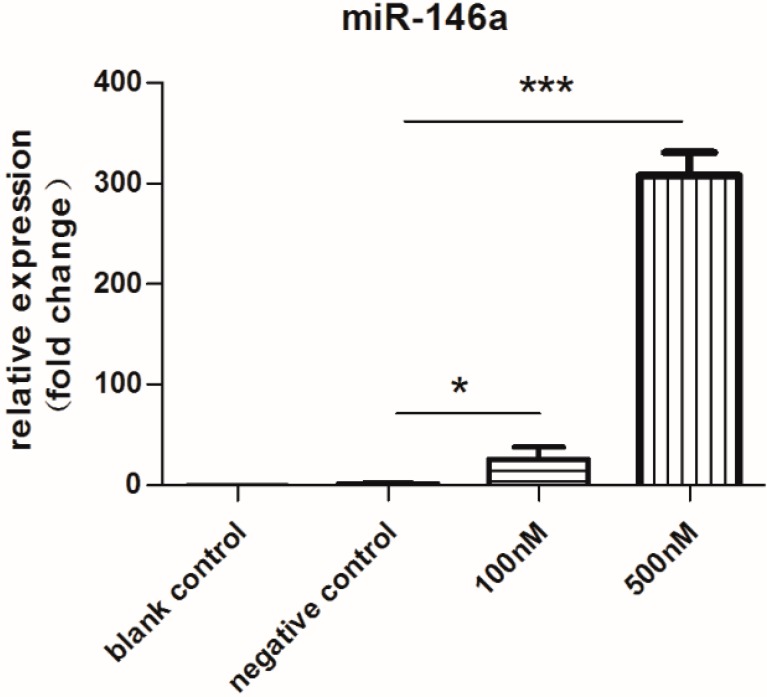
qRT-PCR analysis of miR-146a after 3 days of incubation with MS2 virus-like particles (MS2 VLPs). Cells were stimulated with RANKL/M-CSF for 3 days and treated with 100 or 500 nM MS2-miR-146a VLPs (test groups) or 500 nM MS2-miRNC VLPs (negative control group). Total RNA was extracted from harvested cells using Trizol and stem-loop RT primers were used for reverse transcription. Subsequently, real-time PCR was performed with U6 as an internal reference. The relative expression levels of miRNAs were calculated by the 2^−ΔΔ*C*t^ method. * *p* < 0.05, *** *p* < 0.001. Each experiment was carried out in triplicates.

In this study, PBMCs were treated with 100 or 500 nM MS2-miR-146a VLPs (test groups) or MS2-miRNC VLPs (negative control group). As expected, we observed prominent upregulation of miR-146a in both test groups. Compared with the untreated blank control group, miR-146a expression in the two test groups was increased by 26- and 308-fold, respectively. (*p* < 0.01, Figure 1). Thus, it is clear that this delivery system is capable of upregulating the expression of miR-146a in human PBMCs. Interestingly, the negative control group displayed a 1.5-fold change than that of the blank control.

### 2.2. Expression of Downstream Target Proteins Decreased Dramatically

**Figure 2:**
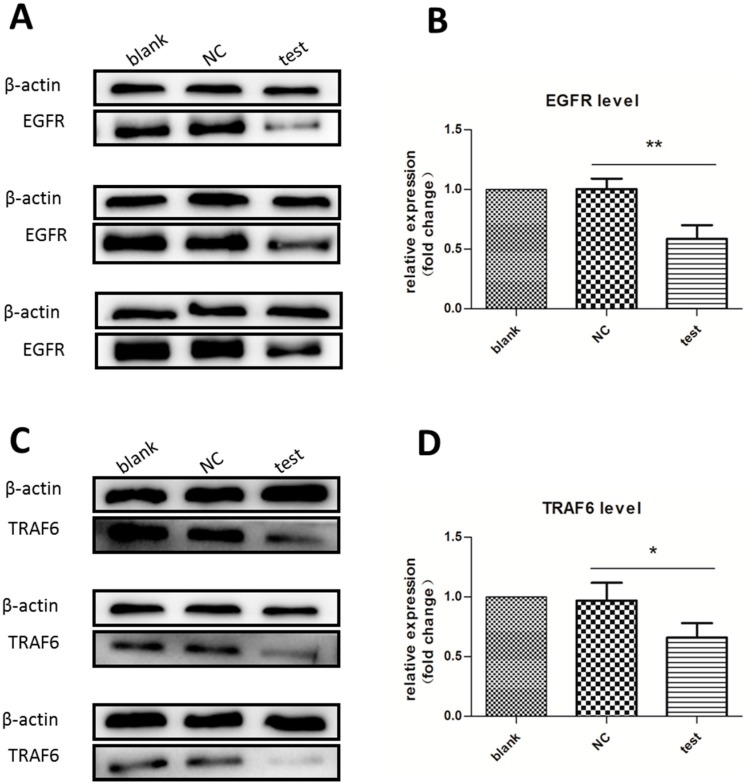
Western blot analysis of EGFR and TRAF6 expression. Cells were stimulated with RANKL/M-CSF for three days and treated with 500 nM MS2-miR-146a VLPs (test group) or 500 nM MS2-miRNC VLPs (negative control group (NC)). Lysates were cleared of cellular debris, and equal concentrations of protein (30 µg) were separated via SDS-PAGE. Proteins were identified by incubating polyvinylidene fluoride (PVDF) membrane with monoclonal antibodies. β-actin was used as a loading control. (**A**,**B**) Expression level of EGFR; and (**C**,**D**) Expression level of TRAF6. * *p* < 0.05, ** *p* < 0.01. Each experiment was carried out in triplicates.

After three days of incubation, the expression of two target proteins, EGFR [18] and TRAF6 [25], were analyzed by Western blot. In this experiment, we did not use two different concentrations of MS2-miR-146a VLP, because preliminary experiments showed that differences in TRAF6 levels in the 100 nM test group were not apparent enough. Compared to the negative control group, the experimental groups treated with MS2-miR-146a VLPs displayed apparently downregulated expression of EGFR and TRAF6 (*p* < 0.01 for EGFR and *p* < 0.05 for TRAF6; Figure 2). However, the difference in expression level between the negative control group and the blank control group was not significant (*p* > 0.05).

### 2.3. Expression of OC-Specific Genes Decreased Rapidly over Time

**Figure 3:**
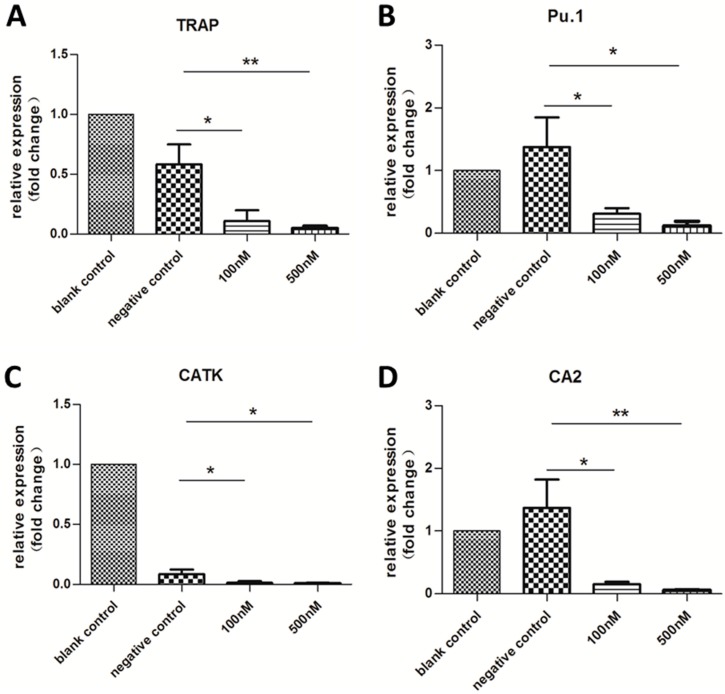
qRT-PCR analysis of OC-specific genes after 14 days of incubation with MS2 VLPs. Cells were stimulated with RANKL/M-CSF and treated with MS2 VLPs. The housekeeping gene glyceraldehyde-3-phosphate dehydrogenase (GAPDH) was chosen as an internal control. (**A**–**D**) represents expression levels of TRAP, Pu.1, CATK, and CA2, respectively. The relative expression levels of miRNAs were calculated by the 2^−ΔΔ*C*t^ method. * *p* < 0.05, ** *p* < 0.01. Each experiment was carried out in triplicates.

After three days of incubation, the expression of the OC-specific genes TRAP [24,26], Pu.1 [27], cathepsin-K (CATK) [24,28], and carbonic anhydrase 2 (CA2) [29] were analyzed by qRT-PCR. However, with the exception of TRAP, expression of these genes was not affected by transfection with MS2-miR-146a VLPs. One of the most important marker genes, Pu.1, was slightly increased (supplementary Figure S1). However, after 14 days of incubation, significantly downregulated expression was observed (*p* < 0.05; Figure 3). Notably, although the level of miR-146a increased in the negative control, the differences in expression of TRAP, Pu.1, and CA2 between blank and negative control groups were not statistically significant (*p* > 0.05). Similarly, even though the expression of miR-146a in the two test groups was different (26- and 308-fold increases), no difference in OC-specific gene expression was detected (*p* > 0.05), indicating that a 26-fold increase in miR-146a is sufficient to regulate the expression of these target genes.

### 2.4. Cytochemical Staining to Characterize OCs

**Figure 4:**
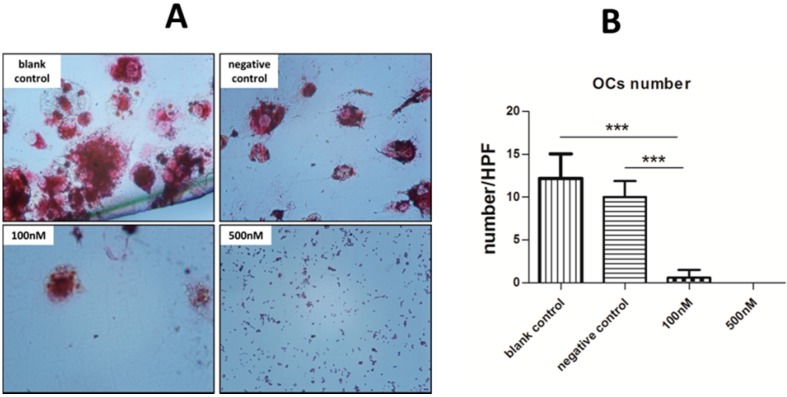
TRAP staining for measurement of the formation of OCs. (**A**) Typical OCs, defined as TRAP-positive and multinucleate cells, were observed by microscopy (original magnification 200×); and (**B**) Five high-power fields were randomly selected for each group and the number of OCs were counted. We then calculated the average number of each group to compare the statistical differences. *** *p* < 0.001. Each experiment was carried out in triplicates.

After 14 days of treatment, adherent cells on glass slides were harvested and cytochemically stained for OC identification. Numerous OCs were formed in the blank control group, but no typical OCs were found in the two experimental groups. The differences between the 100/500 nM groups and the negative control were statistically significant (*p* < 0.001). Furthermore, no differences were observed between the two experimental groups, which concur with the OC-specific gene expression. Unexpectedly, although there were no significant differences in the expression of OC-specific genes between the negative and blank control groups, we found the status of OCs were recognizably different from TRAP staining (Figure 4).

### 2.5. Pit Formation Assay

**Figure 5:**
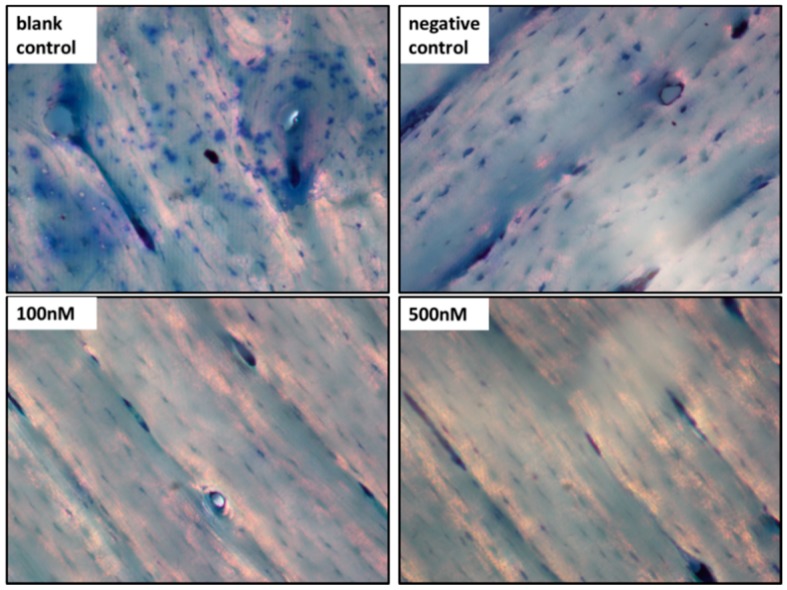
Bone resorption by OCs induced by RANKL and M-CSF. The bone slices were rinsed and left overnight in 1 M ammonium hydroxide to remove all cells. The slices were then washed with PBS and stained with 0.5% toluidine blue. The resorption pits were identified by microscopy (original magnification 200×). Each experiment was carried out in triplicates.

The number of resorptive pits in the blank control group was obviously larger than those of the negative and two experimental groups. More importantly, the number of lacunar resorption pits in the two experimental groups was significantly less than that in the negative control group. We detected no difference in pit formation between the two experimental groups (Figure 5).

## 3. Discussion

As OCs are the major cells responsible for bone resorption, researchers have focused on the inhibition of OC formation to prevent excessive bone loss [17]. MiRNAs, a newly discovered non-coding RNA, have already been used in trials to evaluate their treatment capability. In this study, we focused on the therapeutic ability of one: miR-146a. However, one of the major obstacles is how to choose an appropriate delivery vehicle to ensure that miR-146 expression reaches a suitable concentration to enhance the effect of RNAi. In the present study, we have established an effective method to realize this goal.

Previously, our colleagues developed a novel delivery system to transport miR-146a into human cells. With high efficiency and low toxicity, this system successfully induced overexpression of miR-146a in several human cell lines. We also demonstrated that miR-146a can be an alternative therapeutic strategy for lupus in a BXSB mice model. Meanwhile, this system successfully induced overexpression of miR-146a in PBMCs, and we detected decreased levels of pro-inflammatory cytokines, including Interleukin-6 (IL-6), IL-1β, and TNF-α, in plasma of this model [22]. Herein, we applied this system to human PBMCs and demonstrated inhibition of osteoclastogenesis by upregulated miR-146a.

The MS2 VLP- and Tat-based mechanism was used to increase the level of miR-146a in PBMCs. When PBMCs were transfected with 100 and 500 nM MS2-miR-146a VLPs, miR-146a increased 26- and 308-fold on average, respectively, compared to the blank control group. Thus, this delivery system is capable of inducing an overexpression of miR-146a in PBMCs. Surprisingly, expression of miR-146a in the negative control group, which was treated with MS2-miRNC VLPs, exhibited a 1.5-fold increase. Firstly, one possible interpretation is that the noninfectious MS2 VLPs, as they are genome-free, stimulate strong immune response due to their repeated and ordered surface [30]. Thus, the coat protein of MS2 VLPs, as a heterogeneous stimulus, still has the ability to induce immune response. Coincidently, precursors of OCs belong to the monocyte and macrophage lineage [5], which also exerted a vital role in innate immune response. The coat protein of MS2 VLPs may interact with receptors on the membrance of monocyte and macrophagocyte, causing a nonspecific expression of miR-146a [8]. Secondly, although the level of miR-146a increased in the negative control group, inhibition of osteoclastogenesis was not apparent (as shown in the TRAP results and pit formation assay). This may be because the level of miR-146a not sufficiently high to affect the development of OCs. Thirdly, this phenomenon is consistent with several studies; the constitutive expression of miR-146a is increased in several tissues of RA patients as compared with healthy controls or osteoarthritis patients [10,31,32,33], but the change is too slight to show a detectable influence on osteoclastogenesis. MiRNAs can regulate expression of proteins, but not proteins or cytokines already synthesized and secreted.

Numerous studies have reported the role of M-CSF and RANKL in the differentiation process of OC precursors. RANKL binds with RANK, which belongs to the TNF receptor superfamily, thereby promoting osteoclastogenesis through several signaling transduction pathways. These pathways include the NF-κB pathway and mitogen-activated protein kinase pathways (especially the JNK pathway). Theoretically, modulation of RANK-induced osteoclastogenesis can serve as a therapeutic strategy to control bone loss.

EGFR and TRAF6, the major target proteins of miR-146a, are two crucial signaling factors involved in the RANK crosstalk pathway. EGFR belongs to the ErbB receptor tyrosine kinase family, which includes EGFR/ErbB1, ErbB2/HER2, ErbB3/HER3, and ErbB4/HER4 [34]. RANKL-mediated osteoclastogenesis requires intact EGFR signaling, so EGFR inhibition can block OC differentiation through suppressed activation of JNK and NF-κB [18]. TRAF6 belongs to the TNF receptor superfamily, and is an important adaptor for assembling signaling proteins that are involved in OC-specific gene expression through the NF-κB pathway [24]. In other words, the downregulated TRAF6 level, induced by miR-146a, blocks the signal transduction pathways involved in OC differentiation. In addition, the expression of NF-κB-targeting pro-inflammatory cytokines, including IL-6, IL-1β, and TNF-α, were also suppressed [15,22,32]. Notably, the regulation of TRAF6 for NF-κB is synergistic with EGFR, thus helping to block osteoclastogenesis.

In accordance with qRT-PCR, no typical OCs (TRAP positive and multinuclear) were observed in the two test groups, but numerous OCs were formed in the blank control. What is more, the number of typical OCs in the negative control group was smaller than the blank control group, which was consistent with the result of RT-PCR assay for TRAP and CATK. As noted above, the level of miR-146a in the negative control group slightly increased (Figure 1). Although this small extent of change may not have a significant influence on the formation of OCs or expression of target proteins, it still might delay the process by a few days. In fact, we found a decline in the number of OCs after 14 days of incubation. Moreover, we observed inhibition of OC activity after 21 days of incubation, as shown in the pit formation assay (Figure 5).

Taken together, our results of qRT-PCR assay, Western blot, TRAP staining, and pit formation assay are sufficient for demonstrating the inhibitory role of miR-146a during the formation of OCs.

## 4. Experimental Section

### 4.1. Preparing of Pre-miRNAs Contained MS2 VLPs

The MS2 VLPs containing pre-miR146a were prepared as described previously [21,35]. In brief, the coding sequence of pre-miR146a as well as that of MS2 capsid protein were inserted into the plasmid pACYC-Duet1 (Novagen, Darmstadt, Germany), forming a recombinant plasmid named pMS-miR146. MS2-miR146a VLPs were generated from this recombinant plasmid using *Escherichia coli* BL21 (DE3) expression system and then purified by molecular exclusion chromatography. To achieve effective cell delivery, we conjugated these VLPs with a cell penetrating peptide, an artificially synthesized HIV Tat_47–57_, using the crosslinker sulfosuccinimidyl 4-[*N*-maleimidomethyl] cyclohexane-1-carboxylate (Sulfo-SMCC; Pierce Chemical Co., Rockford, IL, USA). For negative control, another VLP (MS2-miRNC VLPs) was also prepared in the same way using a random sequence instead of pre-miR-146a.

### 4.2. Isolation of PBMCs

Ethics Statement: This study involved PBMCs that originated from human donors. We ensured that an appropriate volume of blood was collected to avoid unnecessary waste. The use of the blood was reviewed and approved by the Ethics Committee of Beijing Hospital. All individual volunteers for this research were informed about the intention of the experiment and the use of the collected blood, before obtaining their signed informed consent. This study does not contain any experiments with animals performed by any of the authors.

The PBMCs were isolated from the peripheral blood of three healthy donors of 26–31 years old with written permission. PBMCs were isolated following the manufacturer’s instructions. Briefly, blood was diluted with phosphate buffered saline (PBS; 1:1) and mixed well. Then, the mixture was added slowly to the top of Human Lymphocyte Separation Medium (Dakewei, Shenzhen, China) in another tube. After centrifugation at 400× *g* for 25 min, the PBMCs layer was recovered and washed twice with PBS.

### 4.3. PBMCs Culture and Transfection with miR-146a or miRNC

We used α-MEM (GIBCO, Invitrogen, Carlsbad, CA, USA), 2 mM l-glutamine, 100 IU/mL penicillin, (GIBCO) and 2.5 µg/mL streptomycin (GIBCO) as the basic medium. After resuspension, the isolated PBMCs were counted and seeded in 24-well culture plates at a concentration of 10^6^ cells per well. PBMCs were cultured at 37 °C and 5% CO_2_ for 4 h. The suspension cells were removed by gentle washing with PBS. The medium was then changed to basic medium plus 10% fetal bovine serum (FBS; GIBCO), 40 ng/mL RANKL (R&D Systems, Minneapolis, MN, USA) [24] and 25 ng/mL M-CSF (R&D Systems) [36,37]. For the negative control group, 500 nM MS2-miRNC VLPs was added, and for the two different test groups, 100 and 500 nM MS2-miR-146a VLPs were added to the medium, respectively. The doses used were based on a previous report [22]. Each experiment was carried out in triplicates. In order to analyze TRAP by cytochemical staining and the bone resorption ability of OCs, glass cover-slips and bovine bone slices were previously placed in corresponding plates.

### 4.4. Quantitative Reverse Transcription Polymerase Chain Reaction (qRT-PCR)

qRT-PCR was performed to evaluate expression of miR-146a and OC-specific marker genes. After 3 days of incubation, total RNA was extracted from harvested cells using Trizol (Invitrogen, Carlsbad, CA, USA) according to the manufacturer’s protocol. For miRNA detection, stem-loop RT primers were designed based on these sequences as previously described (Table 1) [38,39]. The miR-146a expression levels were quantified by real-time PCR using PrimeScript RT reagent Kit (Takara, Shiga, Japan) and SYBR Premix Ex TaqII Kit (Takara). Briefly, small RNAs from each sample were reverse-transcribed to cDNA with stem-loop RT primer and U6 Reverse primer. Subsequently, real-time PCR was performed on StepOnePlus Real-Time PCR System (Applied Biosystems, Foster City, CA, USA) with 95 °C for 30 s, 40 cycles of 95 °C for 5 s and 60 °C for 34 s. Parameters for melting curve analysis (*C*_t_) were 95 °C for 15 s, 60 °C for 1 min, and 95 °C for 15 s. *C*_t_ data was determined by default threshold settings. In this study, U6 RNA was chosen as a miRNA internal control. The relative expression levels of miRNAs were calculated using the 2^−ΔΔ*C*t^ method and the differences in miRNA concentration between treated and control groups were expressed as “fold change” [40].

**Table 1:** Primers used in qRT-PCR Analysis.

Gene	Forward Primers	Reverse Primers
miR-146a	5'-CTAGCTAGCGGCCGCTAGTAACCCATGGAATTCAGTTCTCAG-3'	5'-TCGACTGAGAACTGAATTCCATGGGTTACTAGCGGCCGCTAG-3'
*GADPH*	5'-TGACTTCAACAGCGACACCCA-3'	5'-CACCCTGTTGCTGTAGCCAAA-3'
*TRAP*	5'-CAACGGCTATCTGCGCTTCCA-3'	5'-GAGCTGATCTCCACATAGGCAA-3'
*Pu.1*	5'-GAAGAAGATCCGCCTGTACCAGT-3'	5'-GCCTCCTTGTGCTTGGACGA-3'
*CA2*	5'-GATTCCATTAAAACAAAGGGCAAG-3'	5'-TGAGCACAATCCAGGTCACA-3'
*CATK*	5'-TCCCGCAGTAATGACACC-3'	5'-CCCACAGAGCTAAAAGCCCAA-3'

For OC-specific gene detection, the procedure was similar to that for miR-146a but with two differences: (1) we used oligo (dT) primer and random 6 mers (Takara) instead of the stem-loop RT primers as RT primers; (2) expression of the housekeeping gene glyceraldehyde-3-phosphate dehydrogenase (GAPDH) was chosen as the internal control. Detection was performed 3 and 14 days after MS2 VLPs transfection.

### 4.5. Western Blot Analysis for Downstream Target Proteins

After 3 days of incubation, cells were harvested using trypsin (GIBCO). Cells were lysed in 1× lysis buffer (CST, Danvers, MA, USA) with proteinase inhibitor for 30 min. The supernatant was collected after 8000× *g* centrifuge for 10 min at 4 °C and mixed with 5× SDS loading buffer (Kangweishiji, Beijing, China). After boil denaturation for 5 min, the mixture were electrophoresed on an SDS-PAGE gel, transferred onto a PVDF membrane (0.2 μm; Bio-Rad, Hercules, CA, USA) in a buffer containing 25 mM Tris-HCl (pH 8.3), 192 mM glycine, 20% methanol, and blocked with 5% fat-free dry milk in TBST (Tris-Buffered Saline and Tween 20) for 2 h. These membranes were blotted with anti-EGFR or anti-TRAF6 primary monoclonal antibodies (both from Abcam, Cambridge, UK) and HRP-conjugated goat anti-rabbit/mouse secondary antibodies (Dakewei). And we used SuperSingal West Dura (Thermo Fisher Scientific, Waltham, MA, USA) to play enhanced chemiluminescence (ECL) for blots visualization.

### 4.6. Cytochemical Stain Assay

After 14 days of transfection with MS2 VLPs, cells adhered to the glass slices were cytochemically stained for TRAP using a commercially available kit (Sigma-Aldrich, St. Louis, MO, USA) according to the manufacturer’s protocol. Cells multinucleated and positive for TRAP were identified from three replicas in four independent experiments by light microscopy (Nikon, Tokyo, Japan). We randomly selected five high power fields (HPF) for each group to count the OCs.

### 4.7. Pit Formation Assay

The bone resorption assay was performed 21 days after miRNA administration to monitor OC activity. The bone slices were rinsed with PBS and left overnight in 1 M ammonium hydroxide to remove all cells. The slices were then washed with PBS and stained with 0.5% toluidine blue. The lacunar resorption pits formation was analyzed by light microscopy (Nikon).

### 4.8. Statistical Analysis

All Western blots were quantified with Image J analysis software (National Institutes of Health, Bethesda, MD, USA). Prism 5 software (GraphPad, San Diego, CA, USA) was used for all statistical determinations. Differences between two groups were analyzed by Student’s *t*-tests. Significance was determined as follows: NS *p* > 0.05, * *p* < 0.05, ** *p* < 0.01, *** *p* < 0.001.

## 5. Conclusions

In present study, we used a novel MS2 VLPs-based method to upregulate miR-146a in human PBMCs and observed inhibition of osteoclastogenesis. The results demonstrated that this delivery system was effective for upregulating miR146a in PBMCs, and resulted in dramatic inhibition of osteoclastogenesis and bone resorption. Thus, this approach has potential application in the treatment of osteoporosis.

## Supplementary Materials

Supplementary materials can be found at http://www.mdpi.com/1422-0067/16/04/8337/s1.
