# Supplementary material for: Using a Novel MicroRNA Delivery System to Inhibit Osteoclastogenesis
Source: Int J Mol Sci. 2015 Apr 14;16(4):8337–50. doi: 10.3390/ijms16048337 (PMC4425084; doi:10.3390/ijms16048337)
Supplement: Supplementary file 1 [file ijms-16-08337-s001.pdf]

Supplementary Information

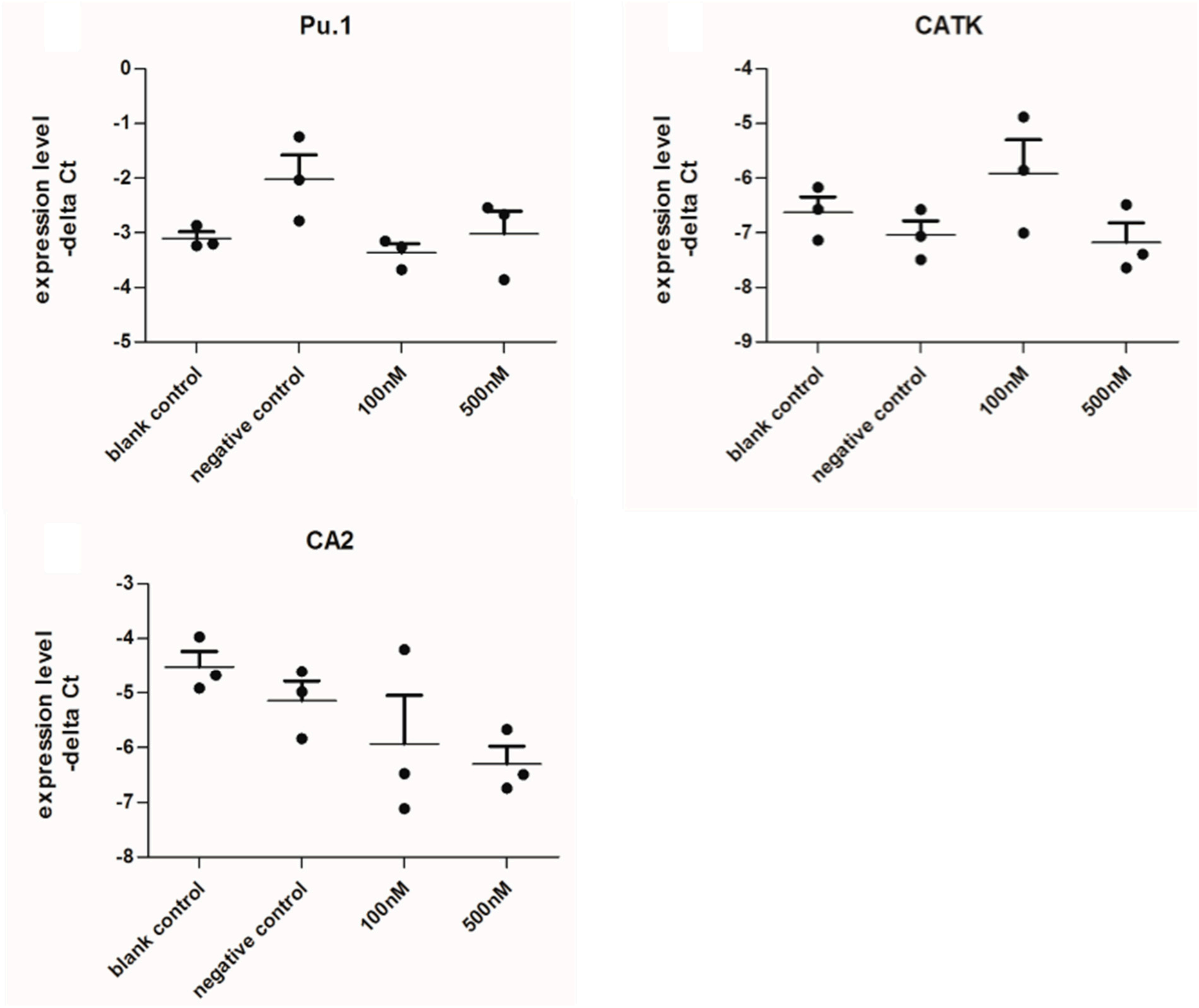

Figure S1. The expression level of osteocalst specified genes after 3 days incubation.
